# A novel real-time PCR assay for specific detection of *Brucella melitensis*

**DOI:** 10.1186/s12879-017-2327-7

**Published:** 2017-03-24

**Authors:** Rene Kaden, Sevinc Ferrari, Erik Alm, Tara Wahab

**Affiliations:** 1 Department of Medical Sciences, Clinical Microbiology, Uppsala University, Uppsala, Sweden; 2 National Veterinary Institute, Uppsala, Sweden; 3 Swedish Forum for Biopreparedness Diagnostics, Stockholm, Umeå and Uppsala, Sweden; 4 Department of Microbiology, The Public Health Agency of Sweden, Stockholm, Sweden

**Keywords:** *Brucella melitensis*, Brucellosis, Real-Time PCR

## Abstract

**Background:**

Brucellosis is a zoonosis that occurs worldwide. The disease has been completely eradicated in livestock in Sweden in 1994, and all cases of confirmed human brucellosis are imported into Sweden from other countries. However, due to an increase in the number of refugees and asylum seekers from the middle-east to Sweden, there is a need to improve the current diagnostic methodology for *Brucella melitensis*. Whilst culture of *Brucella* species can be used as a diagnostic tool, real-time PCR approaches provide a much faster result. The aim of this study was to set up a species-specific real-time PCR for the detection of all biovars of *Brucella melitensis*, which could be used routinely in diagnostic laboratories.

**Methods:**

A *Brucella melitensis* real-time PCR assay was designed using all available genomes in the public database of *Brucella* (*N* = 96) including all complete genomes of *Brucella melitensis* (*N* = 17). The assay was validated with a collection of 37 *Brucella* species reference strains, 120 *Brucella melitensis* human clinical isolates, and 45 clinically relevant non-*Brucella melitensis* strains.

**Results:**

In this study we developed a single real-time PCR for the specific detection of all biovars of *Brucella melitensis*.

**Conclusions:**

This new real-time PCR method shows a high specificity (100%) and a high sensitivity (1.25 GE/μl) and has been implemented in the laboratories of four governmental authorities across Sweden.

## Background

Despite ongoing eradication programs, brucellosis is a widespread zoonosis that infects mainly cattle, sheep, goats, and pigs. It also leads to considerable financial losses in animal husbandry due to abortion and fertility problems in cattle, sheep and goats [1, 2]. Some *Brucella* species can also infect humans and more than 500,000 human cases are reported annually worldwide [3]. Brucellosis is a febrile illness, sometimes with localized bone and tissue infection, or multi-organ disease. Transmission to humans occurs through different routes: the ingestion of unpasteurized milk and dairy products; direct contact with infected animal tissues; or accidental ingestion, inhalation or injection of cultured *Brucella* [4]. Most human Brucellosis cases occur in Syria, Turkey, Mexico and Iran, but also around the Mediterranean basin (Portugal, Spain, Southern France, Italy, Greece, Turkey, North Africa) [3]. *Brucella* comprises six classical species (*B. abortus*, *B. canis, B. melitensis*, *B. neotomae, B. ovis*, and *B. suis*) and five novel species (*B. ceti*, *B. microti, B. inopinata, B. papionis* and *B. pinnipedialis)*. *B. melitensis* is recognized as the main human pathogen associated with human outbreaks worldwide [3, 5, 6]. The species of the genus *Brucella* can be distinguished on the basis of phenotype, genotype and preferred host. Sweden was officially declared free of brucellosis in 1994 [7] and all human cases in Sweden originate from countries abroad. The incidence in Sweden is between 1 and 20 reported cases per year according to the Swedish National Surveillance System SmiNet-2 [8]. The gold standard method for its diagnosis is the isolation of the bacteria from clinical samples via blood cultures and identification by classical microbiological tube testing. *Brucella* grows slowly and visible cultures appear after 3–4 days, but it can take more than 2 weeks to obtain a definitive result [9]. Due to its pathogenicity, a biosafety level 3 laboratory (BSL-3) is mandatory when handling *Brucella* organisms. Laboratory-acquired infections are rarely diagnosed or reported, however they do occur [9, 10].

The application of DNA-based methods for *Brucella* diagnosis is challenging, since all *Brucella* species have a very high degree of genetic homology (up till 99.9%), as shown by whole genome sequencing of *B. abortus*, *B. melitensis*, and *B. suis* [11–14]. However, several groups have recently developed PCR-based assays for the discrimination among species and biovars of *Brucella*. Three *Brucella* species *B. abortus*, *B. melitensis*, and *B. suis* have been sub-typed into biovars [15, 16]. All species within the genus *Brucella* show an average similarity of 99% across the entire genome and a range between 93% and 99.9% based on analysis and nonparametric inference (ANI) analysis. Data from all public available complete genome sequences of all type strains and reference strains were included in the design of primers and probes for the real-time PCR assay of this study. One multiplex PCR (AMOS) developed by Bricker and Halling in 1994, is applicable to differentiate between *B. abortus* biovars 1, 2 and 4, *B. melitensis*, and *B. ovis, B. suis* biovar 1 by specific PCR products based on unique chromosomal loci of the mobile genetic element *IS711* in their genome [17]*.* This PCR was later improved by another laboratory by adding specific primers for the identification of *B. abortus* biovars 5, 6, 9 and genotype 3b of biovar 3 [18]. In 2009, Huber et al. developed a random amplified polymorphic DNA PCR assay to differentiate all recognized *Brucella* species, including the marine mammals-infecting species *B. ceti* and *B. pinnipedialis* [19]. However, all published methods are developed to distinguish species and biovars mainly by gel-based bar patterns and the majority of described methods were tested with a few strains.

Kim et al. [20] developed a new real-time PCR for distinguishing *B. abortus* from other *Brucella* species, which is based on a single nucleotide polymorphism. However, there was a need for a reliable real-time PCR for the detection of *B. melitensis* at the species level [21]. The method has to be validated for clinical purposes with a large number of human isolates to fulfill the validation requirements of the certified laboratory of the public health institute. A *Brucella* genus specific real-time PCR assay is currently in use at the Public Health Agency in Sweden (FOHM). However, *B. melitensis* is the most prevalent species, and we need to be able to differentiate to the species level, due to the epidemiological significance of *B. melitensis* [21].

## Methods

With the aim of developing a real-time PCR assay for the detection of all known species and biovars of *B. melitensis*, the genomes of *B. ceti*, *B. inopinata, B. neotomae* and *B. suis* biovar 4 were sequenced and analyzed. This as necessary due to the lack of necessary genome data in the publicly available databases at the start of this project [14]. The panel to validate the inclusivity and exclusivity of the real-time PCR assay contained all known biovars of *Brucella,* as well as 45 other non-*Brucella* DNA from American Type Culture Collection (ATCC), Culture Collection University of Gothenburg (CCUG) and National Collection of Type Cultures (NCTC) with clinical relevance. A number of environmental samples, as well as the closely related species *Ochrobactrum anthropi,* were also tested in this study (Tables 1 and 2).

**Table 1:** Reference strains tested to assess the sensitivity of *Brucella melitensis* specific real-time PCR assay

Species	Biotype	Strain
*B. melitensis*	1	16 M
	1	ATCC 23456
	1	NCTC 10094
	2	NCTC10508
	3	NCTC 10509
		2065
*B. abortus*	1	ATCC 23448
	1	544
	1	NCTC 00624
	2	NCTC10501
	3	NCTC10502
	4	NCTC10503
	5	NCTC10504
	6	NCTC10505
	7	NCTC10506
	9	NCTC10507
*B. suis*	1	ATCC 23444
	1	NCTC 10316
	1	NCTC 12042–01
	2	NCTC 10510
	3	NCTC 10511
	4	NC 10385–02
	5	NCTC 11996
		1720
		1030
*B. canis*		ATCC 23365
		SVA13
		NCTC 10854
		3.4.2008/122
		E20140122–106
*B. ovis*		ATCC 25840
		NCTC 10512
*B. ceti*		NCTC 12891
*B. inopinata*		CAPM 6436
*B. microti*		CAPM 6434
*B. neotomae*		ATCC 23459
*B. pinnipedialis*		NCTC 12890

**Table 2:** None *Brucella* strains used in the study for exclusivity test

Species	Strain
*Actinobacillus pleuropneumoniae*	CCUG 12837
*Actinomyces pyogenes*	CCUG 13230
*Alcaligenes denitrificans*	CCUG 407
*Bacillus antracis*	NCTC1328
*Bacillus cereus*	CCUG 7414
*Bacillus subtilis*	ATCC 6633
*Bacteroides fragilis*	ATCC 25285
*Bordetella bronchiseptica*	CCUG 219
*Burkholderia mallei*	NCTC120
*Burkholderia pseudomallei*	NCTC8707
*Clostridium perfringens*	CCUG 1795
*Enterococcus fecalis*	ATCC 29212
*Erysipelotrix rhusiopatiae*	CCUG 221
*Escherichia coli*	ATCC 35218
*Escherichia coli (EHEC)*	EDL333
*Escherichia coli (VTEC)*	2954-06
*Fusobacterium necrophorum*	CCUG 9994
*Haemophilus influenzae*	ATCC 49247
*Haemophilus somnus*	CCUG 28029
*Klebsiella oxytoca*	CCUG 15717
*Klebsiella pneumoniae*	CCUG 225
*Listeria monocytogenes*	CCUG 15527
*Nocardia asteroides*	CCUG 10073
*Ochrabactrum anthropi*	ATCC 49188
*Pasteurella multocida*	CCUG 229
*Pasteurella pneumotropica*	CCUG 12398
*Proteus mirabilis*	CCUG 26767
*Pseudomonas aeruginosa*	CCUG 17619
*Rhodococcus equi*	CCUG 892
*Salmonella Dublin*	CCUG 35631
*Salmonella Thyphimurium*	CCUG 31969
*Salmonella Zanzibar*	CCUG 41921
*Staphylococcus aureus*	CCUG 4151
*Staphylococcus intermedius*	CCUG 49053
*Streptobacillus moniliformis*	CCUG 33440
*Streptococcus agalactiae*	CCUG 39325
*Streptococcus dysgalactiae*	CCUG 27436
*Streptococcus equi*	CCUG 27367
*Streptococcus pyogenes*	CCUG 12701
*Streptococcus uberis*	CCUG 27444
*Streptococcus zooepidemicus*	CCUG 23256
*Taylorella equigenitalis*	CCUG 16464
*Yersinia enterocolitica*	CCUG 8239
*Yersinia pestis*	570-04
*Yersinia pseudotuberculosis*	CCUG 5855

### Bacterial strains and growth conditions

A collection of 31 *Brucella* sp. reference strains (Table 1) and 120 *B. melitensis* human clinical isolates were isolated in the BSL-3 laboratory at the FOHM, by cultivation on 5% sheep blood agar plates in a 5%–10% CO_2_ atmosphere at 37 °C for 48 h. All non-*Brucella* strains were routinely cultivated on 5% sheep blood agar over night at 37 °C.

The *B. melitensis* human clinical isolates were collected between 1994 and 2016 from Swedish patients, who had returned from *Brucella*-endemic countries, in a biorepository of the FOHM and used as stipulated in the regulations for diagnostic development and quality assessment. The FOHM performs all *Brucella* diagnostics on human samples in Sweden, and the *B. melitensis* isolates used in this study have previously been confirmed by cultivation, a general real-time PCR amplifying DNA of all *Brucella* strains, as well as by Matrix Assisted Laser Desorption/Ionization Time of Flight Mass Spectrometry (MALDI-TOF MS) [22, 23]. Ethical review of research involving humans is not applicable for diagnostic development and quality assessment.

### DNA extraction

Bacterial DNA was extracted using the commercially available EZ1® DNA Tissue Kit from (Qiagen, Stockholm, Sweden) according to the protocol from the manufacturer and stored at 4 °C until use. A volume of 5 μl of seal herpes virus cell culture was used in each sample of the total volume of 200 μl in the extraction step as a process control. Each sample was eluted in 50 μl elution buffer.

### Bioinformatic analyses, primer & probe design and real-time PCR

In order to identify a *B. melitensis*-specific target(s), all available genomes in the public database of *Brucella* (*N* = 96) including all complete genomes of *B. melitensis* (*N* = 17) were used in the design of primers and probes. A 2 basepair deletion which is highly specific for *B. melitensis* was found in the acetyl-CoA acetyltransferase gene. Primers were designed flanking this deletion and a short 12-mer MGB probe was placed over the area with the deletion. The forward nucleotide sequence 5′-GCTCGACACAAAGGGCCA-3′ (Biomers, Germany) and the reverse nucleotide sequence 5′-CAAGCGTGGTCTGGCGA-3′ (Biomers, Germany) were used with the FAM-labelled hydrolysis probe -CCGAGATACAAA-MGB (Applied Biosystems®).

The real-time PCR assays were carried out in 25 μL reaction mixtures containing 5 μL template DNA, in PerfeCta Multiplex qPCR SuperMix (Applied Biosystems®) diluted in UltraPure™ DNase/RNase-Free Distilled Water (Invitrogen™), 0.9 μM of each primer, and 0.2 μM probe. Amplification and detection were performed using two different PCR machines, an ABI-7500/96-well plates real-time FAST PCR platform, and a StepOne Plus real-time PCR system (Applied Biosystems®).

Thermocycling parameters were as follows: inactivation 95 °C for 3 min, followed by 45 cycles 95 °C for 3 s, and annealing at 60 °C for 30 s. The baseline and threshold were set using the auto-baseline and threshold feature in StepOneplus Software v2.2.2 (Applied Biosystems®). Samples were considered positive if target amplification was detected within 40 cycles.

### Determination of the real-time PCR limit of detection

**Fig. 1:**
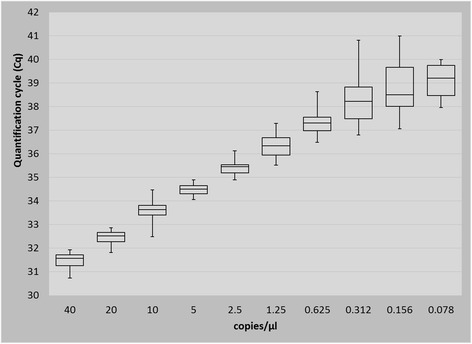
Limit of detection was determined by assaying six replicates of ten and two fold serially diluted DNA of strain *Brucella melitensis* ATCC 23456 in five separate experiments. The number of positives per total number of replicates tested is shown in the figure

The limit of detection (LOD) was defined by using the *Brucella* ATCC 23456 strain with 10^6^, 10^5^, 10^4^, 10^3^, 10^2^, 50, 25, 12.5, 6.25, 3.125, 1.56, 0.78, 0.39 genome equivalents per reaction. LOD samples were analysed in six replicates for each concentration and with five runs on 5 days (Fig. 1).

### Internal amplification control (IAC)

As internal amplification control (IAC), Phocine Herpesvirus 1 (PhHV-1) aliquots with a known DNA concentration and a target quantification cycle (Cq) of 32 was used [24].

## Results and discussion

Real-time PCR is a rapid and reliable method for the analysis of *Brucella* in clinical samples. However, the high genomic similarity between different *Brucella* species makes the design of a species-specific real-time PCR assay difficult. An alternative attempt to use a probe (FAM-CCGCCGAGATACAAA) with Tm 68–70 °C (Primer Express v 3.0) resulted in a signal for all *Brucella* species, but also a signal for non-*melitensis* species. To increase the discriminatory effect, a shorter probe (FAM-CCGAGATACAAA-MGB) with Tm 57 °C was used. By using this shorter probe we could eliminate all the non-specific amplification derived from other *Brucella* species than *B. melitensis*.

The method was validated according to Broeders et al. 2014 [25] and according to the validation standards of the Swedish National Veterinary Institute (SVA), the Public Health Agency of Sweden (FOHM), the Swedish National Food Agency (NFA) and the Swedish Defense Research Agency (FOI). The validation comprised applicability, practicability, specificity, linearity, and sensitivity.

### Applicability

No false positive or false negative result is acceptable in clinical BSL3 pathogen diagnostics. To guarantee a correct identification of BSL3- pathogens, we strongly recommend isolation of the bacteria on selective *Brucella* agar plates. This enables evaluation of the phenotypic properties of the strains, such as colony morphology, and enriches the molecular target of the PCR, as well as reduces the concentration of potential PCR inhibitors. Even after the enrichment an internal PCR process control is recommended as described above. All strains in our validation were cultured under the same conditions as bacterial strains were isolated in the clinical diagnostics workflow. The real-time PCR method was therefore recognized as applicable in combination with the isolation of bacteria from clinical specimens.

### Specificity

The assay was tested with 120 human clinical *B. melitensis* isolates, 31 other non *B. melitensis Brucella* strains, 45 non *B. melitensis* strains, and 6 *B. melitensis* reference strains. There was no amplification from any other *Brucella* species other than *B. melitensis*. The specificity of this method was 100% because all 126 *B. melitensis* samples were tested positive while all 76 non-*B. melitensis* samples gave no amplification in the real-time PCR.

### Practicability

The assay was used for the identification of *Brucella* samples from the first External Quality Assurance Exercises (EQAEs) on highly infectious agents (BSL-3) from the EU financed project “Efficient response to highly dangerous and emerging pathogens” (EMERGE). The results obtained with this assay conformed to the results of other laboratories.

### Sensitivity

The limit of detection was 6.25 genome equivalents of *B. melitensis* (ATCC 23456) per reaction of template DNA (1.25 GE/μl). At a DNA concentration of 3.125 genome equivalents per reaction, 80% of the reactions were still positive (Fig. 1).

### Linearity

The linear dynamic range of this PCR assay was determined by testing serially diluted DNA of *B. melitensis* strain ATCC 23456. The regression coefficient calculated from the regression line in the standard curve was R^2^ = 0.993, y-intercept = 37.766.

In conclusion, the novel real-time PCR assay is highly sensitive and reliable for the rapid detection of all biovars of *B. melitensis*. It is an important tool for diagnostic laboratories for clinical samples from both humans and animals.

## Conclusions

This new real-time PCR method for the detection of all biovars of *B. melitensis* shows a high specificity (100%) and a high sensitivity (1.25 GE/μl) and has been successfully implemented in laboratories of four governmental authorities across Sweden (National Food Agency, National Veterinary Institute, Swedish Defence Research Agency and The Public Health Agency of Sweden).

## Abbreviations

ANI: Analysis and nonparametric inference
ATCC: American type culture collection
B: Brucella
BLAST: Basic local alignment search tool
BSL: Biosafety level
CCUG: Culture Collection University of Göteborg
Cq: Quantification cycle
DNA: Deoxyribonucleic acid
EMERGE: Efficient response to highly dangerous and emerging pathogens
EQAE: External quality assurance exercises
FAM: 6-carboxyfluorescein
FOHM: Public Health Agency of Sweden
IAC: Internal amplification control
LOD: Limit of detection
MALDI-TOF MS: Matrix assisted laser dessorption/Ionization time of flight mass spectrometry
MGB: Minor groove binder
NCBI: National Center for Biotechnology Information
NCTC: National collection of type cultures
NFA: National food agency
PCR: Polymerase chain reaction
PhHV-1: Phocine herpesvirus 1
SVA: National Veterinary Institute
Tm: Melting temperature
